# Inventory of statements about self-harm (ISAS): development and validation of a Chinese short version in a single-center sample

**DOI:** 10.1186/s12888-026-07907-3

**Published:** 2026-03-16

**Authors:** Chuan-Jian Liu, Guo-jiang Wu, Chen Mao, Jun-Jian Xiao, Ling-Yan Li, Shao-Ping Xu, Meng-Xia Xu, Li-Dan Liu, Yang-Li Tian, Xue-Lin Chao, Ai-Lan Wan, Qiao-Sheng Liu, Tao Luo

**Affiliations:** 1 Department of Psychiatry, The First Affiliated Hospital, Nanchang University, Yongwaizheng Street, Donghu District, Nanchang City, Jiangxi Province 330006 China; 2 Student Development Center, Nanchang No.10 Middle School, Nanchang City, Jiangxi Province China; 3 School of Nursing, Jiangxi Medical College, Nanchang University, Nanchang City, Jiangxi Province China; 4 Department of Psychology, Jiangxi Mental Hospital, Nanchang City, Jiangxi Province China

**Keywords:** Inventory of statements about self-harm (ISAS), Non-suicidal self-injury (NSSI), Psychometric validation, Exploratory factor analysis (EFA), Confirmatory factor analysis (CFA), Chinese clinical sample

## Abstract

**Background:**

The Inventory of Statements About Self-harm (ISAS) is a psychometrically sound scale to assess non-suicidal self-injury behavior (NSSI). However, its application in China faces two major constraints: the absence of a validated Chinese version and considerable participant burden due to the scale’s 39-item length. This study aimed to translate the ISAS into Chinese, develop a reduced-item version, and validate its psychometric properties in a clinical sample.

**Methods:**

The ISAS was translated into Chinese and a shortened version of the original scale was developed by theoretical construction and item-total correlation analysis. Psychometric testing was conducted on a clinical sample of 546 patients (age: 18.54 ± 3.86 years) from outpatient and inpatient settings. Reliability was evaluated through the Cronbach’s α coefficient and mean inter-item correlations (MIC). Validity was assessed by examining criterion validity and structural validity, which was evaluated using both exploratory factor analysis (EFA) and confirmatory factor analysis (CFA).

**Results:**

Nearly half of the participants (45.45%) reported engaging in at least one NSSI behavior in the past year. The 13-item Chinese short version of ISAS (ISAS-CS) showed good reliability with favorable Cronbach’s α coefficient and MIC. Criterion validity was confirmed by its significant correlation with the gold standard, the functional assessment of self-mutilation (FASM). Finally, EFA yielded a three-factor model, which was further supported by the subsequent CFA.

**Conclusions:**

This study translated and developed a short-form version of the ISAS, which showed good psychometric properties in a clinical sample. The ISAS-CS can be used as a rapid assessment tool in clinical and research settings.

**Supplementary Information:**

The online version contains supplementary material available at 10.1186/s12888-026-07907-3.

## Background

Non-suicidal self-injury behavior (NSSI) is a severe global health challenge with high prevalence, morbidity, and mortality all over the world [1]. NSSI refers to the deliberate, direct, and self-inflicted destruction of body tissues using some nonlethal methods, such as scratching, burning, and cutting, without an intention to really kill oneself [2]. NSSI has an early onset and typically occurs during childhood and adolescence, a period characterized by increased brain development and hormonal changes, leading to dysregulated emotional control and high-risk behaviors [3]. A recent literature review and meta-analysis on the prevalence of NSSI among adolescents aged 10 to 19 years across 17 countries showed a pooled prevalence of 17.7% in both genders [4]. In addition, female adolescents were twice as likely to have NSSI as male adolescents in North America and Europe, but not in Asia [4]. In China, nearly one in four (24.7%) students aged 5 to 25 reported engaging in NSSI in their lifetime [5], compared to 49.5% in a clinical adolescent sample [6]. The prevalence of NSSI among community adults was 13.4% [7]. Following NSSI, the all-cause mortality increases fourfold, and suicide-related death rises tenfold [4]. This relationship is mediated through mechanisms such as NSSI acting as a “gateway” to suicide by increasing pain tolerance, while both behaviors share significant psychopathological underpinnings and are directly precipitated and moderated by acute life events, particularly interpersonal stressors [4, 8].

The risk factors of NSSI are multiple and can be understood using a biopsychosocial-ecological framework that involves biological (e.g., gender, gene, and immunity), psychological (e.g., personality, psychological disorders, and emotional distress), social (e.g., poor socioeconomic status, poor family environment, and trouble parenting styles), and ecological factors (e.g., migration, discrimination, and toxic interpersonal exposures) [5]. A recent meta-analysis on the risk factors for NSSI in adolescents has identified 80 risk factors grouped into 7 categories: female gender, physical symptoms, low health literacy, adverse childhood experiences, mental disorders, bullying, and problem behaviors [9]. In addition, NSSI predicted a wide range of subsequent adverse clinical outcomes, including mental disorders, substance abuse, sexual risk behaviors, suicide attempts, and committed suicide [10–12]. For instance, Daukantaitė et al. [12] followed up a cohort of 1064 middle school students in Sweden for ten years and found significantly higher levels of stress, anxiety, NSSI, and emotion dysregulation in young adults among those with stable repetitive NSSI in adolescence.

While extensive studies have been conducted to reveal the epidemiological characteristics and trends of NSSI, there are relatively few studies focusing on the prevention and treatment of NSSI [5]. Only a limited number of programs were reported and showed effectiveness in improving clinical outcomes, which encompassed various components (e.g., cognitive behavioral therapy (CBT) and dialectical behavior therapy (DBT)) in multiple formats (e.g., individual therapy, group therapy, and family therapy) [5, 13, 14]. According to a scoping review by Qu et al., a critical research gap exists: the prevalence of NSSI among Chinese adolescents is alarmingly high, yet there is a severe shortage of corresponding, empirically evaluated prevention or intervention programs [5]. The high disease burden of NSSI and limited prevention programs require a valid and reliable assessment tool to evaluate NSSI, which can help guide effective and targeted prevention and intervention programs as well as accurately assess the effectiveness of these programs.

An increasing number of assessment tools have been developed and used to assess NSSI in various samples, including some structured, comprehensive assessment scales, such as the Suicide Attempt Self-Injury Interview (SASII) [15] and Self-Injurious Thoughts and Behaviors Interview (SITBI) [16], scales that are focused on NSSI behaviors such as the Deliberate Self-Harm Inventory (DSHI) [17] and Self-Harm Inventory (SHI) [18], and self-report scales focused on NSSI functioning such as the Functional Assessment of Self-Mutilation (FASM) [19] and the Inventory of Statements About Self-harm (ISAS) [20]. Consequently, various literature reviews have been conducted to summarize and compare the psychometric properties of the various self-report scales for NSSI [21–25]. A recent systematic review of instruments up to 2020 has identified 26 validated instruments for NSSI out of 109 possible instruments for self-injury. Most NSSI scales were self-reported English scales focusing on NSSI function and topography, developed and validated predominantly in North America [25]. Despite the large number and diversity of NSSI scales, there is only limited evidence on the reliability and validity [25].

Among the various NSSI self-report scales, FASM and ISAS have demonstrated good psychometric properties in the assessment of NSSI functions based on NSSI behaviors. However, FASM lacks the evaluation of essential NSSI functions such as sensation seeking, coping with suicidal thoughts, and interpersonal boundaries [20]. In comparison, ISAS is more comprehensive, encompassing both the behaviors and functions of NSSI [20]. The ISAS was developed by Klonsky & Glenn based on a non-clinical college student sample [20]. It assesses thirteen function domains under two major categories: intrapersonal (including affect regulation, anti-dissociation, anti-suicide, marking distress, and self-punishment) and interpersonal (including autonomy, toughness, revenge, self-care, sensation seeking, peer bonding, interpersonal influence, and interpersonal boundaries). The initial psychometric testing of the ISAS showed high α coefficients and test-retest correlations for both categories [20, 26, 30].

Since the ISAS was initially developed with a non-clinical sample in the US, cross-culture validation and adaptation in various populations are needed for its broad application. So far, the ISAS has been translated into multiple languages, including Spanish [27], Turkish [28], Swedish [29] and Urdu [30]. However, it has not been translated into Chinese and validated in a Chinese context. In addition, the full version of ISAS is lengthy and contains 39 items, which may increase the burden of respondents and lead to measurement errors, especially in a clinical sample with emotional instability. The advantage of a culturally adaptive short form of the ISAS lies in its ease of administration and minimal respondent burden, which renders it particularly valuable for quick screening in high-volume clinical and research settings [28]. Therefore, this study aimed to translate and develop a brief Chinese version of ISAS and validate it in a Chinese clinical sample.

## Methods

### Study design, participants, and procedure

A cross-sectional study was conducted by recruting patients with mental and behavior disorders from the outpatient and inpatient departments of Jiangxi Mental Hospital from February 2022 to December 2022. The sample size was determined based on established guidelines for scale validation [31], adhering to the principle of a minimum 10:1 participant-to-item ratio and a total *N* ≥ 200 for EFA. With 13 items in our scale, we employed a more stringent 20:1 ratio to ensure a more stable factor structure and greater statistical power, resulting in a preliminary minimum of 260 participants. We then adjusted this target to 289 per group to account for an estimated 10% of invalid questionnaires and the need to split the sample for both EFA and CFA, establishing a final total sample target of 578.

Inclusion criteria included: (1) participants who met the ICD-10 diagnostic criteria for mental and behavioral disorders [32]; (2) participants aged between 14 and 60 years; and (3) participants who had one or more NSSI behaviors in the past year, as identified by a psychiatrist based on patient self-reports, family reports, and physical examination. We excluded those who were unable to complete the questionnaire due to communication barriers, low literacy, severe cognitive impairment, or severe physical impairment.

This study was approved by the Internal Review Board (IRB) of Jiangxi Mental Hospital (No.:20190112). Potential participants were approached by the department nurses, and those interested in the study were referred to our research team. Before the study began, the research team explained the study’s purpose and procedure to the participants and their families. The participants were fully informed that participation in the study was voluntary, and they could withdraw from the study at any time, which would not affect their treatment at the hospital. After physicians confirmed the presence of NSSI behavior through clinical interviews and physical examinations, eligible patients were enrolled in the study and administered the questionnaire survey. All participants’ personal information was kept strictly confidential. After signing the informed consent form, each participant was invited to complete a structured questionnaire.

### Measurement

#### Demographics questionnaire

A researcher-designed Demographics Questionnaire was used to collect the participants’ demographic information such as age, gender, education, and employment.

#### ISAS

The ISAS scale was compiled by Klonsky and Glenn [20] and consists of two sections to measure NSSI-related behavior frequencies and potential functions, respectively. The first section includes 12 items asking about the lifetime frequencies of 12 NSSI behaviors and the context in which they occurred. The 12 behaviors are as follows: cutting, biting, burning, carving, pinching, pulling hair, severe scratching, banging or hitting self, interfering with wound healing, rubbing skin against the rough surface, sticking self with needles, and swallowing dangerous substances. In addition, six more questions are asked about the age of first NSSI, the time of last NSSI, the duration between self-injury ideation and behavior, whether they were alone, whether they felt pain, and whether they thought about stopping it. The second section comprises 39 items that assess 13 potential functions of NSSI, with each function being measured by 3 items. These functions include: affect regulation, interpersonal boundaries, self-punishment, self-care, anti-dissociation, anti-suicide, sensation seeking, peer-bonding, interpersonal influence, toughness, marking distress, revenge, and autonomy. Each item is rated on a three-point Likert score with the following options: 0 (not relevant), 1 (somewhat relevant), and 2 (very relevant). The total dimension score of each function ranges from 0 to 6 points. The 13 functions are divided into a two-factor structure, namely interpersonal functions and intrapersonal functions [20]. This structure was further supported in subsequent validations [24].

#### Translation of the ISAS

The translation of the ISAS involves the following three steps: (1) Forward translation. The original English version of the ISAS was translated into Chinese by a Ph. D student in psychiatry, who was proficient in Chinese and English and familiar with both cultures. Cultural acceptability and conceptual clarity were given priority during the translation process. (2) Back translation. The translated Chinese version of the ISAS was back-translated into the English version by two independent bilingual Master students in Psychology who were not exposed to the original English version of the ISAS. (3) Cross-language validation. The back-translated English version of the ISAS was compared with the original English version by another Ph. D student who was not involved in the previous translation and back-translation processes. To establish semantic and conceptual equivalence, an expert panel comprising one psychology specialist and two psychiatry experts was convened to review and refine all ambiguous or poorly phrased items. The final Chinese version was subsequently confirmed for its translation accuracy, ensuring the original sentence structures and meanings remained intact.

#### Development of the ISAS short-form

The process for developing the ISAS short-form version (ISAS-CS) followed three main stages:

1. We formed a committee of 10 experts with multidisciplinary backgrounds in psychiatry, psychology, clinical medicine, psychotherapy, and psychiatric nursing. The core task of the committee was to assess the quality of the original scale items, with a primary focus on their representativeness within their respective 13 domains and the clarity of their phrasing. The assessment utilized a quantitative 0–10 point scoring system. Based on the scores, items with higher expert consensus were prioritized to form a preliminary item pool.
2. To ensure the applicability of the scale for the target population, we subsequently conducted one-on-one interviews with 20 patients who had a history of NSSI. Through these interviews, we assessed the comprehensibility and psychological impact of each item from the perspective of the patients. The sample size of 20 was chosen to achieve information saturation in qualitative analysis, thereby obtaining diverse and in-depth feedback.
3. We established a clear mechanism for re-evaluation: if an item was reported by three or more respondents to have significant comprehension difficulties, it would be resubmitted to the expert committee for discussion and revision. Taking the “Emotion Regulation” domain as an example, the item “alleviating anxiety, frustration, anger, or other unbearable emotions” received the highest score in the initial expert evaluation. However, during patient interviews, 10 respondents (50% of the sample) provided feedback that the phrasing was too verbose and suggested that “calming myself down” was more concise and closer to everyday language. Based on this critical user feedback, the expert committee decided in subsequent discussions to revise the item to “calming myself down” and include it in the short-form scale.

#### Functional assessment of self-mutilation (FASM)

FASM was designed by Lloyd et al. [19] and consists of two sections to measure NSSI-related behaviors and functions, respectively. The first section includes 11 items measuring the frequency and characteristics of 11 NSSI behaviors under two categories based on severity: moderate/severe (cutting, burning, erasing the skin, and self-tattooing) and mild (pulling hair, inserting objects under nails or skin, biting self, hitting self, picking at a wound, scratching skin, self-punching). For each positive behavior, there are five more questions asking about the degree of physical pain, the amount of time they thought about engaging in SMB, and the use of alcohol or drugs during self-injury. The second section includes 22 items measuring functional motivations of NSSI under four dimensions, corresponding to the four-function model proposed by Nock (2009), namely autonomous positive reinforcement (APR), autonomous negative reinforcement (ANR), social positive reinforcement (SPR), and social negative reinforcement (SNR). The scale uses a four-point Likert scale, ranging from 0 to 3, corresponding to “never,” “occasionally,” “sometimes,” and “often,” respectively. In China, Leong et al. [33] first revised it into Chinese and modified the functional motivation to 21 items, which has shown good psychometric properties in subsequent validation testing. In the current study, the Chinese version of FASM showed good internal consistency with a Cronbach’s α coefficient of 0.69 [95% confidence interval (CI): 0.65, 0.73].

#### Patient health questionnaire-9 scale (PHQ-9)

PHQ-9 is a self-rating depression scale that meets the DSM-IV diagnostic criteria for depressive disorders [34]. It includes a total of 9 items scored on a four-point Likert scale ranging from 0 to 3 points, corresponding to “none,” “a few days,” “more than half the time,” and “almost every day,” respectively. The total score ranges from 0 to 27 points, with a higher score indicating more depressive symptoms. This scale assesses the frequency of subjects’ depressive symptoms in the past two weeks and has good reliability and validity when applied to the Chinese population [35, 36]. In the current study, the PHQ-9 showed good internal consistency, with a Cronbach’s α of 0.80 (95% CI: 0.78, 0.82).

#### Generalized anxiety disorder-7 scale (GAD-7)

The GAD-7 is a self-rating scale used to screen and assess anxiety disorders [37]. It contains a total of 7 items scored on a four-point Likert scale ranging from 0 to 3, corresponding to “none,” “a few days,” “more than half the time,” and “almost every day,” respectively. The total score ranges from 0 to 21 points, with a higher score indicating more anxiety symptoms. This scale assesses the frequency of subjects’ anxiety symptoms in the past two weeks. Like the PHQ-9, the GAD-7 also shows good reliability and validity in the Chinese [38, 39]. In the current study, the GAD-7 showed good internal consistency, with a Cronbach’s α of 0.83 (95% CI: 0.80, 0.85).

#### McLean screening instrument for borderline personality disorder (MSI-BPD)

The MSI-BPD is a self-administrated scale developed by Zanarini et al. [40] based on the diagnostic criteria for borderline personality disorder in DSM-IV. It contains a total of 10 dichotomized items with optional answers of “yes” (1 point) and “no” (0 points). The total score ranges from 0 to 10 points, with a higher score indicating a higher tendency for BPD. This scale has shown good reliability and validity in clinical applications in China [41]. In the current study, the MSI-BPD showed good internal consistency, with a Cronbach’s α of 0.76 (95% CI: 0.73, 0.79).

#### Rosenberg self-esteem scale (RSES)

The Rosenberg Self-Esteem Scale (RSES) is a self-rating scale that is widely used to assess individual self-respect and self-acceptance [42]. It contains 10 items in total, with 5 positively worded items and 5 negatively worded items using reverse scoring. Each item is rated on a four-point Likert scale ranging from 1 (totally disagree) to 4 (totally agree). The total score ranges from 10 to 40 points, with a higher score indicating a higher level of self-esteem. The RSES has been widely used in the Chinese population with good reliability and validity. In the current study, the RSES showed acceptable internal consistency, with a Cronbach’s α of 0.60 (95% CI: 0.55, 0.65).

### Statistical analysis

#### Data processing and analysis

All statistical analyses were performed using SPSS 25.0 and Mplus 8.3, with the significance level set at α = 0.05. The dataset contained a very low rate of missing data (< 0.5%). In line with contemporary methodological recommendations for handling missing data in structural equation modeling, we employed the Full Information Maximum Likelihood (FIML) estimation in Mplus. Descriptive statistics were employed to summarize the sample’s demographic and clinical characteristics. Continuous variables were presented as mean (standard deviation, SD) if they were normally distributed, or as median (interquartile range, IQR) if they were non-normally distributed. The normality of distribution was assessed using the Shapiro-Wilk test and visual inspection of histograms. Categorical variables were presented as frequencies and percentages (N, %). This reporting follows the statistical analyses and methods in the published literature (SAMPL) guidelines for statistical reporting [43, 44]. For data that did not meet the assumption of normal distribution, non-parametric statistical methods were applied. Descriptive statistics, item analysis, reliability testing, exploratory factor analysis, and preliminary correlation analyses were performed with SPSS. Confirmatory factor analysis was subsequently carried out using Mplus.

#### Reliability and validity testing

The strength of association between each item and the total scale score was examined using Spearman rank correlation analysis. The internal consistency reliability of the scale was assessed using Cronbach’s α coefficient (with > 0.70 considered acceptable) and the mean inter-item correlation (acceptable range: 0.15–0.50) [45]. In terms of validity, criterion validity was tested by calculating Spearman rank correlations between the ISAS and FASM, PHQ-9, GAD-7, MSI-BPD, and RSES.

#### Factor analysis

To further assess the structural validity of the scale, the 546 participants were randomly divided into two independent subsamples at a 1:1 ratio (*n* = 273 for each) for use in exploratory factor analysis (EFA) and confirmatory factor analysis (CFA), respectively. EFA was performed using principal axis factoring with Promax oblique rotation, based on a polychoric correlation matrix to accommodate the ordered categorical nature of the data. Factors were retained according to the criteria of eigenvalues greater than 1 and factor loadings of no less than 0.4 [46]. For CFA, the weighted least squares means and variance adjusted (WLSMV) method was selected for parameter estimation, as it is suitable for ordinal data and does not assume multivariate normality. To further examine the robustness of the model, we conducted an Exploratory Structural Equation Modeling (ESEM) analysis using the full sample. Model fit was evaluated using the following indices: χ2/df (relative/normed chi-square) < 5 (Wheaton, 1987), RMSEA (root mean square error of approximation) ≤ 0.08, CFI (comparative fit index) ≥ 0.90, Tucker-Lewis index (TLI) ≥ 0.90, and SRMR (standardized residual mean root) ≤ 0.08 [47, 48].

## Results

### Sociodemographic and clinical characteristics of the sample

Following clinical interviews and physical examinations of 1,208 patients, 549 (45.45%) were identified and enrolled in the questionnaire survey. After excluding 3 invalid questionnaires with inconsistent or missing answers, a total of 546 valid questionnaires were obtained, with an effective rate of 99.5%. The sample comprised 546 participants with a mean age of 18.54 ± 3.86 years. It was characterized by a high concentration (92.12%, 503/546) of individuals aged 16–20 years, representing a population of late adolescents and early adults. Additionally, the sample was predominantly female (83.9%), unmarried (96.2%), and currently students (71.4%). Major depressive disorder (41.9%) and bipolar disorder (36.3%) were the most common diagnoses (Table 1).

**Table 1:** Sociodemographic and clinical characteristics of the sample

Parameter	Male (*n* = 88)	Female (*n* = 458)	Total (*n* = 546)
	Median (IQR) / *N* (%)	Median (IQR) / *N* (%)	Median (IQR) / *N* (%)
Age (Year)	18 (17, 18)	18 (18, 20)	18 (17, 19)
Educational Level			
Primary or below	2 (2.3)	1 (0.2)	3 (0.5)
Secondary	52 (59.1)	315 (68.8)	367 (67.2)
University or above	34 (38.6)	142 (31.0)	176 (32.2)
Marital Status			
Unmarried	80 (90.9)	451 (98.5)	525 (96.2)
Married	8 (9.1)	7 (1.5)	21 (3.8)
Employment status			
Full-time employment	14 (15.9)	60 (13.1)	74 (13.6)
Part-time employment	0 (0.0)	22 (4.8)	22 (4.0)
Unemployed	17 (19.3)	43 (9.4)	60 (11.0)
In education	57 (64.8)	333 (72.7)	390 (71.4)
Mental disorder diagnoses			
Major depressive disorder	43 (48.9)	186 (40.6)	229 (41.9)
Bipolar disorder	22 (25.0)	176 (38.4)	198 (36.3)
Schizophrenia spectrum disorder	16 (18.2)	72 (15.7)	88 (16.1)
Other mental disorders	7 (8.0)	24 (5.2)	31 (5.7)

### Characteristics of NSSI

The lifetime frequency of non-suicidal self-injury (NSSI) among participants was 16.65 ± 16.13 episodes. Among the 546 participants, 533 (97.60%) reported more than one episode of NSSI in the past year, and 506 (92.67%) reported five or more episodes. Furthermore, the vast majority (94.5%) experienced their first NSSI episode before the age of 18. There were no significant gender differences in the frequency of NSSI behaviors, but the age of first self-injury was significantly lower in females than in males (Table S1).

Table 2 shows the frequencies and characteristics of the 12 NSSI behaviors. The most common NSSI behavior was cutting (78.2%), followed by banging or hitting (55.1%) and pinching (29.5%). The most common duration from NSSI ideation to behaviors was within one hour (46.3%), followed by between 1 and 3 h (26.6%). Most of the participants had their last NSSI behaviors within six months (96.3%) and committed NSSI when they were alone (88.5%). After performing NSSI behaviors, 59.7% felt pain, and 73.8% thought about stopping.

**Table 2:** Frequency and characteristics of NSSI behaviors (*n* = 546)

Variables	Frequency (Proportion, %)	Median (IQR)	Corrected item-total correlation	Cronbach’s α if item deleted
NSSI Behaviors				
Cutting	427 (78.2)	5 (1, 5)	0.539^**^	0.764
Biting	142 (26.0)	0 (0, 1)	0.601^**^	0.702
Burning	117 (21.4)	0 (0, 0)	0.483^**^	0.717
Carving	74 (13.6)	0 (0, 0)	0.652^**^	0.698
Pinching	161 (29.5)	0 (0, 1)	0.469^**^	0.718
Pulling hair	148 (27.1)	0 (0, 1)	0.539^**^	0.715
Severe scratching	126 (23.1)	0 (0, 0)	0.551^**^	0.709
Banging or hitting self	301 (55.1)	1 (1, 5)	0.714^**^	0.684
Interfering wound healing	128 (23.4)	0 (0, 0)	0.445^**^	0.720
Rubbing skin	158 (28.9)	0 (0, 1)	0.413^**^	0.724
Sticking self with needles	40 (7.3)	0 (0, 0)	0.503^**^	0.718
Swallowing dangerous substances	100 (18.3)	0 (0, 0)	0.319^**^	0.732
Other	106 (19.4)	0 (0, 0)	0.352^**^	0.728
Duration between self-injury ideation to behavior				
<1	253 (46.3)			
1–3 h	145 (26.6)			
3–6 h	19 (3.5)			
6–12 h	13 (2.4)			
12–24 h	28 (5.1)			
>24 h	88 (16.1)			
Time of the last NSSI				
Within 6 moths	526 (96.3)			
After 6 months	20 (3.7)			
Whether being alone during NSSI				
No	63 (11.5)			
Yes	483 (88.5)			
Whether feeling pain				
No	220 (40.3)			
Yes	326 (59.7)			
Whether thinking of stopping				
No	143 (26.2)			
Yes	403 (73.8)			

Note: ** *p*<0.01. NSSI: Non-suicidal self-injury behavior

### Item-total analysis

The original Inventory of Statements about Self-Injury (ISAS) functional scale is composed of 13 domains, with each domain containing three items. We compared the Spearman rank correlation of each item with the total score after the item was deleted (the sum of the scores of the remaining 38 items). The item-total analysis supported the theoretical construction of the short form, confirming that the items selected for inclusion were those with the highest item-total correlation coefficients within their respective functional domains [49]. as shown in Table 3.

**Table 3:** The correlation between each item and the total score after its deletion

Functions	Items	The correlation	Cronbach’s α if item deleted	Cronbach’s α if item deleted in short version
Affect regulation	When I self-harm, I am …			
	**1. calming myself down**	0.52^**^	0.940	0.909
	14. releasing emotional pressure that has built up inside of me	0.41^**^	0.941	
	27. reducing anxiety, frustration, anger, or other overwhelming emotions	0.36^**^	0.941	
Interpersonal boundaries	**2. creating a boundary between myself and others**	0.63^**^	0.939	0.900
	15. demonstrating that I am separate from other people	0.50^**^	0.940	
	28. establishing a barrier between myself and others	0.50^**^	0.940	
Self-punishment	**3. punishing myself**	0.54^**^	0.940	0.907
	16. expressing anger towards myself for being worthless or stupid	0.41^**^	0.941	
	29. reacting to feeling unhappy with myself or disgusted with myself	0.44^**^	0.941	
Self-care	**4. giving myself a way to care for myself (by attending to the wound)**	0.44^**^	0.940	0.906
	17. creating a physical injury that is easier to care for than my emotional distress	0.40^**^	0.940	
	30. allowing myself to focus on treating the injury, which can be gratifying or satisfying	0.46^**^	0.940	
Anti-dissociation	**5. causing pain so I will stop feeling numb**	0.66^**^	0.939	0.898
	18. trying to feel something (as opposed to nothing) even if it is physical pain	0.66^**^	0.939	
	31. making sure I am still alive when I don’t feel real	0.59^**^	0.939	
Anti-suicide	**6. avoiding the impulse to attempt suicide**	0.51^**^	0.940	0.905
	19. responding to suicidal thoughts without actually attempting suicide	0.45^**^	0.940	
	32. putting a stop to suicidal thoughts	0.51^**^	0.940	
Sensation seeking	**7. doing something to generate excitement or exhilaration**	0.49^**^	0.939	0.903
	20. entertaining myself or others by doing something extreme	0.33^**^	0.940	
	33. pushing my limits in a manner akin to skydiving or other extreme activities	0.36^**^	0.940	
Peer-bonding	8. bonding with peers	0.41^**^	0.941	
	**21. fitting in with others**	0.60^**^	0.939	0.904
	34. creating a sign of friendship or kinship with friends or loved ones	0.34^**^	0.941	
Interpersonal influence	9. Let others know the extent of my emotional pain	0.64^**^	0.938	
	**22. seeking care or help from others**	0.71^**^	0.938	0.897
	35. keeping a loved one from leaving or abandoning me	0.48^**^	0.940	
Toughness	10. seeing if I can stand the pain	0.44^**^	0.939	
	23. demonstrating I am tough or strong	0.44^**^	0.939	
	**36. proving I can take the physical pain**	0.54^**^	0.939	0.902
Marking distress	11. creating a physical sign that I feel awful	0.36^**^	0.940	
	**24. proving to myself that my emotional pain is real**	0.46^**^	0.940	0.903
	37. signifying the emotional distress I’m experiencing	0.44^**^	0.940	
Revenge	**12. getting back at someone**	0.54^**^	0.939	0.902
	25. getting revenge against others	0.37^**^	0.940	
	38. trying to hurt someone close to me	0.45^**^	0.940	
Autonomy	**13. ensuring that I am self-sufficient**	0.45^**^	0.940	0.906
	26. demonstrating that I do not need to rely on others for help	0.35^**^	0.940	
	39. establishing that I am autonomous/independent	0.32^**^	0.940	

Note: ** *p*<0.01. Items in bold were retained in the Chinese Short version of the NSSI

### Reliability

The reliability of the ISAS-CS was tested by measuring the Cronbach’s α coefficient and MIC of each item and dimension (see Table S2). The Cronbach’s α coefficient was 0.73 for the behavioral dimension and 0.89 for the functional dimension. The correlations of the 13 behavioral items and the total behavioral dimension ranged from 0.32 to 0.71, all of which were significant. The MIC value was 0.22 for the behavioral dimension and 0.37 for the functional dimension. All these results suggest the ISAS-CS has acceptable reliability.

### Validity

The ISAS-CS showed significant correlations with established measures related to self-injury and mental health. Both the behavioral and functional dimensions were positively correlated with the FASM behavioral and functional dimensions. Furthermore, they were positively associated with depression, anxiety, and borderline personality traits, and negatively associated with self-esteem (Table 4).

**Table 4:** The correlations between ISAS-CS and FASM, PHQ-9, GAD-7, MSI-BPD, and RSES

Measures	FASM behaviors	FASM functions	PHQ-9	GAD-7	MSI-BPD	RSES
ISAS-CS behaviors	0.79^**^	0.49^**^	0.45^**^	0.49^**^	0.25^**^	-0.33^**^
ISAS-CS functions	0.42^**^	0.44^**^	0.31^**^	0.34^**^	0.23^**^	-0.20^**^
Alleviating negative feelings	0.39^**^	0.35^**^	0.34^**^	0.37^**^	0.20^**^	-0.15^**^
Regulating interpersonal relationships	0.43^**^	0.38^**^	0.24^**^	0.29^**^	0.28^**^	-0.22^**^
Inducing positive feelings	0.20^**^	0.36^**^	0.12^**^	0.11^**^	0.12^**^	-0.14^**^

Note: ** *p*<0.01. ISAS-CS: the Chinese short version of Inventory of Statements About Self-harm. FASM: Functional Assessment of Self-Mutilation Scale. PHQ-9: Patient Health Questionnaire-9 Scale. GAD-7: Generalized Anxiety Disorder-7 Scale. MSI-BD: McLean Screening Instrument for Borderline Personality Disorder. RSES: Rosenberg Self-Esteem Scale

When examining the three-factor structure of the functional dimension, the “Alleviating negative feelings” subscale demonstrated the highest correlation with both depression (*r* = 0.34) and anxiety (*r* = 0.37). In comparison, the “Regulating interpersonal relationships” subscale showed the strongest associations with borderline personality traits (*r* = 0.28) and self-esteem (*r* = -0.22).

### EFA

The participants were randomly divided into two equal groups for EFA and CFA, respectively. EFA was used to explore the underlying factor structure of the ISAS-CS functional dimension. The KMO value was 0.837, and the P value of Barrett’s sphericity test was < 0.001, indicating the scale was suitable for factor analysis. Previous studies have shown significant correlations between the different items of the ISAS functional dimension [20], and our study also showed similar results. Therefore, principal axis factoring and Promax rotation in SPSS were used to extract and rotate factors to examine the factorial structure of the 13 items contained in the ISAS-CS functional dimension. The EFA produced three initial eigenvalues above 1, thus yielding a three-factor solution that accounted for 65.5% of the variance (Table 5 and Figure S1), which was inconsistent with the original two-factor structure proposed by Klonsky and Glenn [20].

In order to test whether the changes in the structure were caused by the short version of the scale, we ran another EFA on the original full-item ISAS functional dimension, and the results showed the same three-factor structure. A careful comparison between the original two-factor structure and the new three-factor structure showed that the intrapersonal factor remained unchanged, while the original interpersonal factor was divided into two factors, thus forming a three-factor structure. According to the characteristics of the items included in each factor, we named these three factors as follows: alleviating negative feelings (the original intrapersonal factor), regulating interpersonal relationships, and inducing positive feelings. The three factors showed high Cronbach’s α coefficients (0.83, 0.86, and 0.80) and were positively correlated with the total functional dimension (*r* = 0.84, *r* = 0.84, *r* = 0.73, *P* < 0.01 for all).

**Table 5:** Factor loadings of the ISAS-CS using EFA

Items		3-factor structure	
	Alleviating negative feelings (factor1)	Regulating interpersonal relationships (factor2)	Inducing positive feelings (factor3)
Interpersonal influence		0.967	
Affect regulation	0.935		
Autonomy			0.885
Self-care			0.840
Revenge		0.777	
Anti-dissociation	0.708		
Peer-bonding		0.701	
Interpersonal boundaries		0.658	
Self-punishment	0.641		
Marking distress	0.602		
Toughness			0.596
Sensation seeking			0.565
Anti-suicide	0.553		

Note: ISAS-CS: Chinese Short version of Inventory of Statements About Self-injury function. EFA: exploratory factor analysis

### CFA

**Fig. 1:**
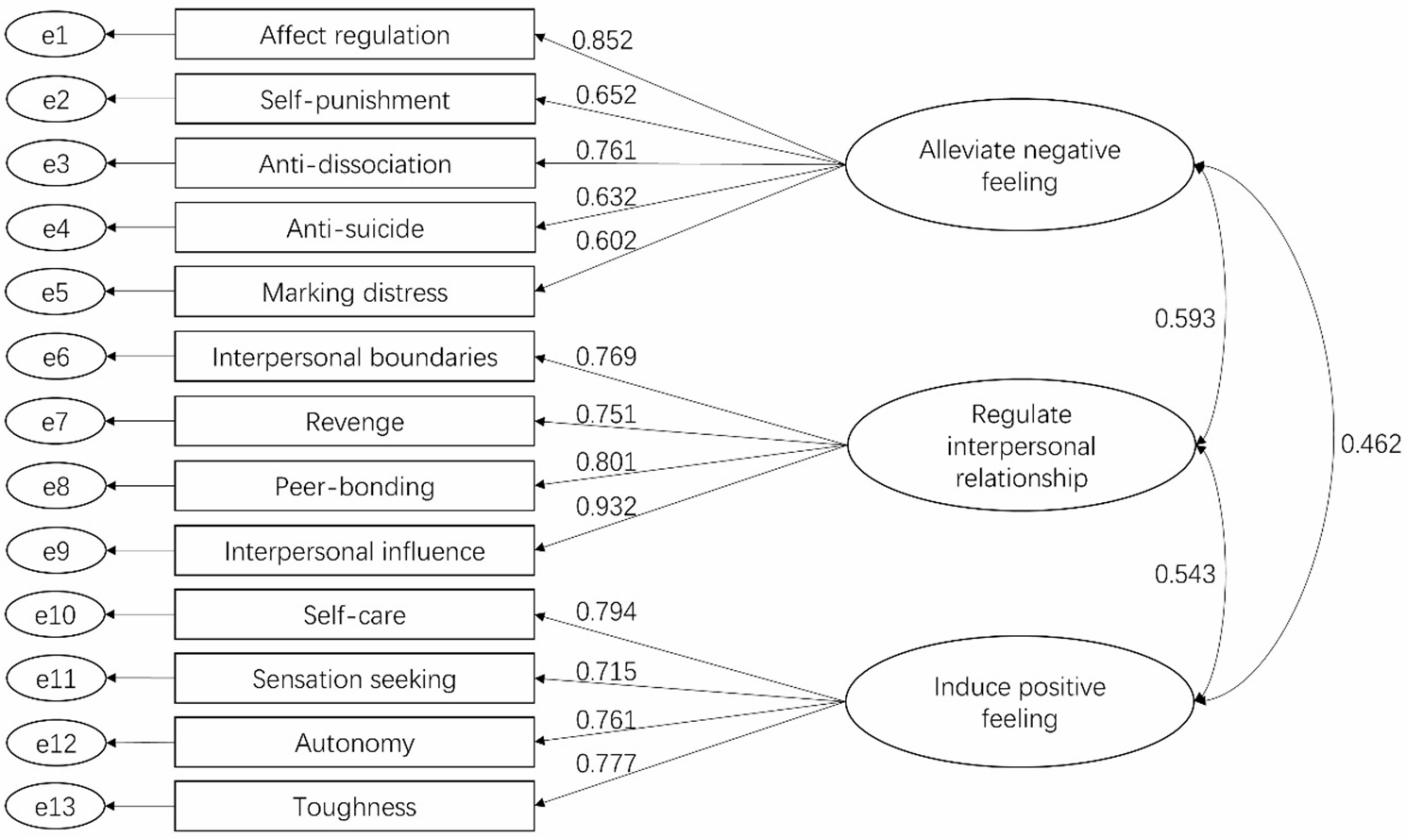
Factor structure of the three-factor model for the ISAS-CS. Note: ISAS-CS: Chinese Short version of Inventory of Statements About Self-injury

CFA: Confirmatory factor analysis; χ2/df: relative/normed chi-square; RMSEA: root mean square error of approximation; CFI: comparative fit index; TLI: Tucker-Lewis index; SRMR: standardized residual mean root.

CFA is used to verify the theoretical structure derived from EFA to find out whether the theoretical structure is established. Using the second half of the sample, we conducted CFA to compare the model fits of three different factor structures: a single-factor model, the original two-factor model, the new three-factor model. As shown in Table 6, both the single-factor model and the original two-factor model showed poor model fit. In contrast, the new three-factor model showed the best model fit (Fig. 1), indicating good structural validity.

**Table 6:** Model fit index of the three-factor structures of ISAS-CS using CFA

	χ^2^/df	RMSEA	CFI	TLI	SRMR
1-factor	10.453	0.186	0.663	0.596	0.112
2-factor	7.409	0.153	0.775	0.726	0.098
3-factor	2.787	0.081	0.943	0.926	0.061

Note: ISAS-CS: Chinese Short version of Inventory of Statements About Self-injury function

### ESEM

To rigorously validate the robustness of the three-factor model, we employed ESEM, an innovative methodological approach that merges the complementary strengths of EFA and CFA. As articulated by Marsh et al., ESEM integrates EFA’s flexibility in empirically delineating underlying factor structures with the advanced statistical rigor inherent in CFA [50]. This unique combination allows for a more precise and nuanced specification of latent constructs, addressing limitations of traditional EFA (which lacks confirmatory testing) and CFA (which relies on pre-specified factor structures that may not align with empirical data). The ESEM results supported the three-factor structure, with good model fit indices: χ²/df = 4.80, RMSEA = 0.083, CFI = 0.958, TLI = 0.922, SRMR = 0.026. All target loadings were statistically significant, and no substantial cross-loadings were observed (Table S3). Thereby providing additional statistical support for the current three-factor model.

## Discussion

### Summary of the findings

NSSI is a highly prevalent and disabling disorder that negatively affects individuals, families, and society, posing a significant disease burden worldwide [5, 11]. A psychometrically sound measure of NSSI is essential not only for the diagnosis and assessment but also for the development of future evidence-based prevention and intervention programs and evaluation of their effectiveness. In this study, we translated a widely used NSSI assessment tool, ISAS, and then revised its 39-item functional domain to develop its first Chinese short-form version (ISAS-CS), reducing it to 13 items through theoretical construction and item analysis.

The ISAS-CS was further validated in a clinical sample of Chinese patients with mental and behavioral disorders. Our results showed a high prevalence of NSSI, with 45.45% of patients reporting at least one NSSI behavior in the past year. The ISAS-CS showed high reliability, which is reflected in its high Cronbach’s α coefficients and satisfactory MIC values. Criterion validity was confirmed by its significant correlation with the FASM, PHQ-9, GAD-7, MSI-BPD, and RSES. The sample was further randomly divided into two equal groups for EFA and CFA, respectively, to explore and verify the factor structure of the functional dimension of the ISAS-CS. An EFA yielded a three-factor model: alleviating negative feeling, regulating interpersonal relationship, and inducing positive feeling, which was further supported by the subsequent CFA with good model fit. Thus, the ISAS-CS demonstrated psychometrically sound properties for assessing NSSI among Chinese patients with mental and behavioral disorders.

### Characteristics of NSSI

In this study, up to 45.45% of patients with mental and behavioral disorders reported at least one NSSI behavior in the past year, with the three most common NSSI behaviors being cutting, banging or hitting, and pinching. This finding was consistent with previous studies reporting the frequencies and methods of NSSI among various populations in various countries [1, 4, 5, 10]. Qu et al. [5] conducted a literature review of 256 studies up to 2022 across 28 provinces of China and reported a pooled life prevalence of 24.7% for NSSI among the Chinese youth population aged 5 to 25, with common methods including scratching, hitting, and biting. Our study showed a higher prevalence of NSSI in the clinical sample than in the general population and similar patterns of NSSI methods between the clinical and community samples.

In addition, the profile of participants who engaged in NSSI indicates that the typical individual was under the age of 18 at the time of their first self-injury, often engaged in NSSI while alone, exhibited repetitive NSSI behaviors, and had their most recent episode within the past six months. These findings align with previous research indicating that individuals with mental disorders are highly susceptible to NSSI, which frequently emerges during early adolescence. Our sample was predominantly composed of young, unmarried females (83.9%), with a mean age of 18.54 years, and the majority of participants were still in education (71.4%). The most common diagnoses were major depressive disorder (41.9%) and bipolar disorder (36.3%). These demographic and clinical characteristics may have collectively influenced the observed patterns of NSSI, particularly the early onset feature. Our findings suggest that NSSI is a prevalent and serious challenge among adolescents, highlighting the necessity and urgency of early diagnosis and timely intervention. A comprehensive understanding of the functional motivation of individuals committing NSSI can help develop more effective NSSI prevention and intervention programs [51, 52].

### Reliability of the ISAS-CS

The reliability of the ISAS-CS was tested by calculating the Cronbach’s α coefficient and mean inter-item correlations of the scale. Cronbach’s α coefficients for the behavioral and functional dimensions both exceeded 0.70, indicating good internal consistency reliability. This finding is congruent with previous studies showing high Cronbach’s α s for the full ISAS scale in non-Chinese samples by previous studies [43, 53]. Although Cronbach’s α coefficients are sensitive to the number of items and decrease with reduced items [54], the short version revealed Cronbach’s α coefficients similar to the full version, further demonstrating item homogeneity and internal consistency of the ISAS-CS. The MIC is another indicator of internal consistency, and it measures the extent to which one item’s score is related to scores on all other items on a scale [55]. The ideal MIC value ranges from 0.15 to 0.50, and a much lower MIC indicates that the items may not represent the same construct, while a much higher MIC suggests that the items may not capture the full construct [55]. Our study showed that the MIC values are within the acceptable range, indicating a balance between item homogeneity and uniqueness.

### Validity of the ISAS-CS

The criterion validity of the ISAS-CS was tested by calculating its correlation with the gold standard—the FASM. Our results showed that both the behavioral and functional dimensions of the ISAS-CS were positively and significantly correlated with the FASM and its dimensions, thus confirming the criterion validity of the ISAS-CS. In addition, the ISAS-CS was significant positive correlations with PHQ-9, GAD-7, and MSI-BPD, as well as its significant negative correlation with the RSES. These findings were in line with the extensive literature showing that individuals with higher NSSI frequency and stronger NSSI motivation have more depressive and anxiety symptoms, a higher tendency towards borderline personality disorder, and lower self-esteem [56–58]. These findings support our conclusion that the ISAS-CS has good validity, with strong correlations with other similar concepts.

### Factor structure of the ISAS-CS

The factor structure of the functional dimension of the ISAS-CS was tested through a multi-step analytic strategy. We first used EFA on one randomly split half-sample to explore the underlying structure, which yielded a three-factor model. This model was then confirmed using CFA on the second half-sample, demonstrating better fit than alternative one- and two-factor models. To further examine the robustness of this structure, we conducted an ESEM analysis on the full sample, which allows for cross-loadings and provides a more flexible test of dimensionality. The ESEM results supported the same three-factor structure, with good model fit indices. This convergent evidence from EFA, CFA, and ESEM strongly supports the three-factor model, which differs from the original two-factor structure reported in previous research [20]. Compared to the two-factor structures of interpersonal and intrapersonal factors, the three-factor structure retained the original intrapersonal factor, renamed as alleviating negative feelings. In contrast, the original interpersonal factor was divided into two factors: regulating interpersonal relationships and inducing positive feelings. This new model is more consistent with a clinical sample of patients with mental and behavioral disorders in a Chinese context. In previous studies, many researchers proposed that the main function of NSSI was to regulate emotions within an individual, that is, intrapersonal functions [52, 59]. Our study showed that alleviating negative feelings, regulating interpersonal relationships, and inducing positive feelings were all significantly and positively correlated with the ISAS functional total score, with similar *r* values. These findings suggest that the interpersonal functions of NSSI are equally important as the intrapersonal functions and should not be ignored [60]. The difference may stem from cultural variations between our Chinese sample and the Western populations that existing NSSI models are based on. In collectivist cultures, NSSI may serve more as a coping mechanism for interpersonal stress, such as academic competition, peer conflict, or family expectations [61].

### Limitations

Although this study demonstrated good reliability and validity of the ISAS-CS, some possible limitations should also be considered. First, the ISAS-CS was validated in a clinical sample of Chinese patients with mental and behavioral disorders in a hospital, and which may not represent other populations, such as non-clinical populations in the community. Additionally, our sample exhibited limited heterogeneity in key demographic characteristics, being predominantly composed of young, unmarried, and educated females. This sample profile may reflect two practical realities: first, the clinical prevalence of NSSI is relatively higher in adolescents and early young adults; second, individuals in this age group generally have fewer concerns about disclosing self-injurious behaviors and demonstrate stronger willingness to participate in related research. Consequently, the generalizability of the findings to older adult clinical populations requires further investigation. Future multi-center studies are needed to validate the scale in other populations with various diagnoses and in different areas. Second, the cross-sectional study design cannot test the scale’s sensitivity to change. Future longitudinal studies are warranted to test whether its sensitivity changes over time or after interventions. Third, the test-retest reliability of the ISAS-CS cannot be evaluated since no retest has been done on this sample. Future studies should conduct a retest to expand the reliability of the scale.

## Conclusions

This study translated and developed a Chinese short version of ISAS (ISAS-CS) and confirmed its reliability and validity for a clinical sample of Chinese people with mental and behavioral disorders. The ISAS-CS inherits the accurate and comprehensive characteristics of the original scale to the greatest extent while greatly reducing the number of questions and respondent burden, which minimizes measurement errors caused by respondent fatigue and promotes measurement efficiency. The validation of the ISAS-CS allows for its use as an alternative to the full ISAS as a rapid assessment tool in clinical and research settings in a Chinese context. Further validation with other populations and in other countries is warranted in the future.

## Supplementary Information

Below is the link to the electronic supplementary material.

Supplementary Material 1

## Data Availability

The datasets used are available from the corresponding author upon reasonable request.

## Abbreviations

NSSI: Non-Suicidal Self-Injury
ISAS: Inventory of Statements About Self-injury function
ISAS-CS: the Chinese short version of Inventory of Statements About Self-harm
FASM: Functional Assessment of Self-Mutilation Scale
PHQ-9: Patient Health Questionnaire-9 Scale
GAD-7: Generalized Anxiety Disorder-7 Scale
MSI-BD: McLean Screening Instrument for Borderline Personality Disorder
RSES: Rosenberg Self-Esteem Scale
CFA: Confirmatory factor analysis
RMSEA: root mean square error of approximation
CFI: comparative fit index
TLI: Tucker-Lewis index
SRMR: standardized residual mean root
WLSMV: the weighted least squares means and variance adjusted
SAMPL: the statistical analyses and methods in the published literature
FIML: Full Information Maximum Likelihood
ESEM: Exploratory Structural Equation Modeling
