# Supplementary material for: Inventory of statements about self-harm (ISAS): development and validation of a Chinese short version in a single-center sample
Source: BMC Psychiatry. 2026 Mar 16;26:340. doi: 10.1186/s12888-026-07907-3 (PMC13104386; doi:10.1186/s12888-026-07907-3)
Supplement: Supplementary file 1 — Supplementary Material 1 [file 12888_2026_7907_MOESM1_ESM.pdf]

Additional files information

Table S1. Gender differences in non-suicidal self-injury (n=546)

Table S2. Internal Consistency of ISAS-CS, FASM, PHQ-9, GAD-7, MSI-BPD, and RSES

Table S3. Exploratory Structural Equation Modeling factor loadings for the ISAS-CS.

Figure S1. Scree Plot for the exploratory factor analysis of the Chinese Short version of ISAS functions

Additional files

Table S1. Gender differences in non-suicidal self-injury (n=546)

		Median	P25, P27	Mann-Whitney U（Sig.）
NSSI frequency	Male	10	5, 18	0.058
	Female	10	6, 24	
First NSSI	Male	14.20	13, 15	0.000**
	Female	13	13, 15	

Note: $^{**}p < 0.01$.

Table S2. Internal Consistency of ISAS-CS, FASM, PHQ-9, GAD-7, MSI-BPD, and RSES

Measures	Median (P25, P27)	Cronbach's α	MIC
ISAS-CS behaviors	10 (6, 22)	0.728	0.22
ISAS-CS functions	3 (1, 8)	0.886	0.37
FASM behaviors	10 (5, 19)	0.614	0.12
FASM functions	10 (7, 14)	0.688	0.12
PHQ-9	11 (8, 18)	0.800	0.38
GAD-7	10 (6, 15)	0.825	0.44

MSI-BPD	6 (3, 7)	0.761	0.25
RSES	21 (17, 24)	0.598	0.15

Note: ISAS-CS: the Chinese Short version of Inventory of Statements About Self-harm. FASM: Functional Assessment of Self-Mutilation Scale. PHQ-9: Patient Health Questionnaire-9 Scale. GAD-7: Generalized Anxiety Disorder-7 Scale. MSI-BD: McLean Screening Instrument for Borderline Personality Disorder. RSES: Rosenberg Self-Esteem Scale.

Table S3. Exploratory Structural Equation Modeling factor loadings for the ISAS-CS.

Item	Factor 1	Factor 2	Factor3
Affect regulation	0.615 (p<0.001)	-0.016 (p=0.498)	0.090 (p=0.091)
Interpersonal boundaries	0.005 (p=0.900)	0.768 (p<0.001)	0.045 (p=0.304)
Self-punishment	0.584 (p<0.001)	0.100 (p=0.336)	0.010 (p=0.828)
Self-care	-0.151 (p=0.003)	0.006 (p=0.042)	0.934 (p<0.001)
Anti-dissociation	0.586 (p<0.001)	0.342 (p<0.001)	0.002 (p=0.921)
Anti-suicide	0.637 (p<0.001)	0.158(p=0.144)	-0.008 (p=0.806)
Sensation seeking	0.086 (p=0.162)	0.279 (p<0.001)	0.433(p<0.001)
Peer-bonding	0.105 (p=0.118)	0.623 (p<0.001)	-0.028 (p=0.561)
Interpersonal influence	0.038 (p=0.544)	0.836 (p<0.001)	-0.004 (p=0.911)
Toughness	-0.013 (p=0.697)	0.370 (p<0.001)	0.451 (p<0.001)
Marking distress	0.331 (p<0.001)	0.210 (p=0.010)	0.244 (p<0.001)
Revenge	-0.217 (p=0.002)	0.935 (p<0.001)	0.006 (p=0.668)
Autonomy	0.006 (p=0.628)	-0.157 (p=0.013)	0.953 (p<0.001)

Note: ISAS-CS: the Chinese Short version of Inventory of Statements About Self-harm.

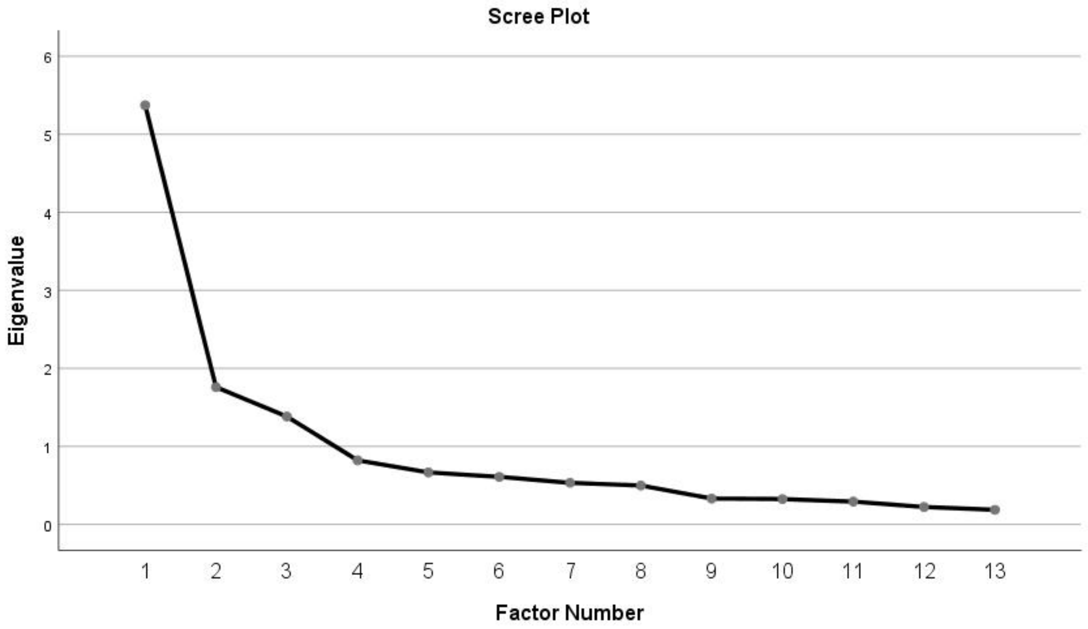

Figure S1. Scree Plot for the exploratory factor analysis of the Chinese Short version of ISAS functions
